# Physical Exercise Restores the Generation of Newborn Neurons in an Animal Model of Chronic Epilepsy

**DOI:** 10.3389/fnins.2017.00098

**Published:** 2017-03-01

**Authors:** Fabricio N. Mendonça, Luiz E. C. Santos, Antônio M. Rodrigues, Sérgio Gomes da Silva, Ricardo M. Arida, Gilcélio A. da Silveira, Fulvio A. Scorza, Antônio-Carlos G. Almeida

**Affiliations:** ^1^Laboratório de Neurociência Experimental e Computacional, Departamento de Engenharia de Biossistemas, Universidade Federal de São João del-ReiSão João del-Rei, Brazil; ^2^Instituto do Cérebro, Hospital Israelita Albert EinsteinSão Paulo, Brazil; ^3^Núcleo de Pesquisas Tecnológicas, Universidade de Mogi das CruzesMogi das Cruzes, Brazil; ^4^Departamento de Fisiologia, Escola Paulista de Medicina, Universidade Federal de São Paulo (UNIFESP)São Paulo, Brazil; ^5^Disciplina de Neurologia Experimental, Escola Paulista de Medicina, Universidade Federal de São Paulo (UNIFESP)São Paulo, Brazil

**Keywords:** epilepsy, physical exercise, neurogenesis, ectopic newborn neurons, DCX

## Abstract

Neurogenesis impairment is associated with the chronic phase of the epilepsy in humans and also observed in animal models. Recent studies with animal models have shown that physical exercise is capable of improving neurogenesis in adult subjects, alleviating cognitive impairment and depression. Here, we show that there is a reduction in the generation of newborn granule cells in the dentate gyrus of adult rats subjected to a chronic model of epilepsy during the *postnatal* period of brain development. We also show that the physical exercise was capable to restore the number of newborn granule cells in this animals to the level observed in the control group. Notably, a larger number of newborn granule cells exhibiting morphological characteristics indicative of correct targeting into the hippocampal circuitry and the absence of basal dendrite projections was also observed in the epileptic animals subjected to physical exercise compared to the epileptic animals. The results described here could represent a positive interference of the physical exercise on the neurogenesis process in subjects with chronic epilepsy. The results may also help to reinterpret the benefits of the physical exercise in alleviating symptoms of depression and cognitive dysfunction.

## Introduction

Temporal lobe epilepsy (TLE) is a neurological disorder characterized by the occurrence of spontaneous and recurrent seizures (Fisher et al., 2005; Duncan et al., 2006). Epilepsy has been associated with cognitive dysfunction and depression (Hattiangady and Shetty, 2008). Among the epilepsy-related alterations in brain function are the loss of hippocampal functional inhibition, the reorganization of hippocampal circuitry and neurodegeneration (Hattiangady and Shetty, 2008, 2010). In the last years, abnormal neurogenesis in the dentate gyrus has been one of the main topics of focus in TLE investigations (Parent et al., 1997, 2006; Scharfman et al., 2000, 2002, 2003; Parent and Lowenstein, 2002; Hattiangady et al., 2004; Kuruba et al., 2009; Hattiangady and Shetty, 2010). In animal models of epileptogenesis, the first moments after *status epilepticus* (SE) (latent phase) are characterized by increased cellular proliferation and pronounced aberrant neurogenesis. However, the chronic phase of the disease is marked by a substantial reduction in neurogenesis (Hattiangady et al., 2004; Walter et al., 2007; Kuruba et al., 2009).

In addition to the decrease in neurogenesis in the dentate gyrus, the ectopic migration of granule cells (aberrant neurogenesis) has also been observed in brain slices from epileptic patients (Parent et al., 2006) as well as in experimental models of chronic epilepsy (Parent et al., 1997; Scharfman et al., 2000; Dashtipour et al., 2003; Bonde et al., 2006; Jessberger et al., 2007a,b; Walter et al., 2007). It has been proposed that these ectopic granule cells act as “*hub cells*,” receiving a disproportional amount of excitatory input compared with normotopic granule cells and being able to generate intrinsic bursts, trigger seizures and, therefore, play a significant role in epileptogenesis (Scharfman and Pierce, 2012; Hester and Danzer, 2013). An additional abnormality described in experimental models of epilepsy is the occurrence of granule cells with basal dendrites with aberrant projections into the hilus. This phenomenon and the ectopic granule cells have been considered enough to increase excitability due to the formation of recurrent circuits connecting the mossy fibers and the normotopic granule cells of the dentate gyrus (Ribak et al., 2000; Austin and Buckmaster, 2004; Shapiro and Ribak, 2006; Hattiangady and Shetty, 2008; Thind et al., 2008).

In mammals, the subgranular zone of the dentate gyrus is one of the two brain areas where neurogenesis is present throughout life (Gage, 2000; Alvarez-Buylla and Lim, 2004; Duan et al., 2008; Zhao et al., 2008; Ma et al., 2009; Ming and Song, 2011). Evidence has shown that physical exercise can induce hippocampal neurogenesis by increasing the release of neurotrophins, particularly brain-derived neurotrophic factor (BDNF) (Cotman and Berchtold, 2002; Vaynman et al., 2004; Vaynman and Gomez-Pinilla, 2005; Olson et al., 2006). Therefore, physical exercise has been proposed as potential non-invasive complementary therapy promoting health and nervous tissue repair in distinct neurological/neurodegenerative syndromes (Cotman and Berchtold, 2002; Russo-Neustadt et al., 2004; Cotman et al., 2007; van Praag, 2008; Arida et al., 2013; Intlekofer and Cotman, 2013). In the present work, we investigated the effect of physical exercise on the number, location, and morphology of newborn neurons within the dentate gyrus in adult rats subjected to SE during the *postnatal* period of brain development. Notably, physical exercise was able to restore the generation of newborn neurons to the level observed in the control group. Despite the continued existence of ectopic newborn neurons, an increase on the number of newly generated granule cells exhibiting morphological characteristics indicating normal migration and integration into the hippocampal circuitry were observed in the epileptic rats subjected to the physical exercise.

## Materials and methods

### Animals

All procedures involving animals were approved by the Institutional Animal Care and Use Committee guidelines from the Federal University of São João del-Rei, and all efforts were made to minimize animal suffering and to reduce the number of animals used. Water and food were freely available, and room humidity (21 ± 2°C) and temperature (50 ± 10%) were controlled and the animals were housed in a 12:12 h light-dark cycle.

### Status epilepticus induction and epileptogenesis

According to Cavalheiro et al. (1987), the maturity of the brain cholinergic neurons is required so that the *status epilepticus* can trigger the epileptogenic process, which will lead to the development of chronic TLE. Therefore, to attain functionally mature cholinergic neurons (Cavalheiro et al., 1987), 28-day-old male Wistar rats (*N* = 50) were injected with pilocarpine chloride (320 mg/kg, i.p.) to induce SE. Prior to the pilocarpine injection, rats were injected with methylscopolamine (1 mg/kg, i.p.) to preclude peripheral damage. After 120 min of SE, rats were given an injection of diazepam (10 mg/kg, i.p.) to mitigate seizure activity. The rats that survived SE (*N* = 16) were monitored 24 h a day by a motion detection system and infrared night illumination from day 45 to *postnatal* day 59, with all the animals having at least two spontaneous seizures (with a minimum interval of 24 h between seizures) since the last day of monitoring. The following groups of rats were formed after 30 days of SE: SE—rats solely subjected to SE (*N* = 8); SE/EX—rats subjected to SE with subsequent physical exercise (*N* = 8) and C—control rats (*N* = 8).

### Training procedure

Animals in all groups were familiarized with treadmill running and the trainability test using the scale proposed by Dishman et al. (1988). Literature data indicate that 3 days of exercise are able to increase the level of neurotrophins and cell proliferation in the hippocampus (Gómez-Pinilla et al., 1997; van der Borght et al., 2009) and 7 days of running on the treadmill are able to increase the number of young DCX^+^ neurons (Brown et al., 2003; Steiner et al., 2004; Uda et al., 2006; van der Borght et al., 2009). The expression of doublecortin (DCX) occurs in a transient state of neuronal development that lasts for approximately 2 weeks (Kim et al., 2002). In the present study, we chose to use a protocol of physical exercises where the initial phase of increase in both proliferation and generation of newborn neurons were stabilized. Thus, at the end of the physical exercise protocol, the neurons belonging to the proliferation of the first days of physical exercise would already be adults and no longer expressing DCX, however, due to the continuing physical exercise, other newborn neurons will be taking place and the DCX expressing level would be stabilized at an increased but constant level. Then, after the familiarization process, the rats in the SE/EX group were subjected to 28 sessions of treadmill running over the course of 4 weeks (1 session/day). A warm-up consisting of running at a speed of 8 m/min for 5 min preceded all exercise sessions. The speed and duration of each session were 10 m/min and 10 min, increasing in increments of 2 m/min and 5 min per week to a maximum of 16 m/min and 25 min in the last week. The rats in the other two groups were subjected to all the processes associated with transport to the exercise room and the corresponding manipulation, but were not subjected to the physical exercise session.

### Immunohistochemistry

At 90 days of age, the rats were anesthetized with an overdose of ketamine-xylazine (100–10 mg/kg, respectively) followed by transcardial perfusion with 0.1 M phosphate-buffered saline (PBS; pH 7.4) followed by a 2% paraformaldehyde (PFA) fixative solution. The brains were dissected, post-fixed in PFA for 24 h, washed, and stored in PBS at 4°C until sectioning. Brains were sectioned coronally at a thickness of 40 μm with a vibrating microtome (Leica Microsystems, Wetzlar, Germany). The histological sections were preincubated for 90 min at room temperature in blocking solution (10% BSA and 0.1% Triton X-100). Overnight incubation in the primary antibody solution containing 2% BSA was subsequently performed. An anti-DCX antibody (rabbit polyclonal, 1:1,000; Abcam, Cambridge, USA) was used to stain developing immature neurons.

### Confocal microscopy and histological analyses

**Figure 1:**
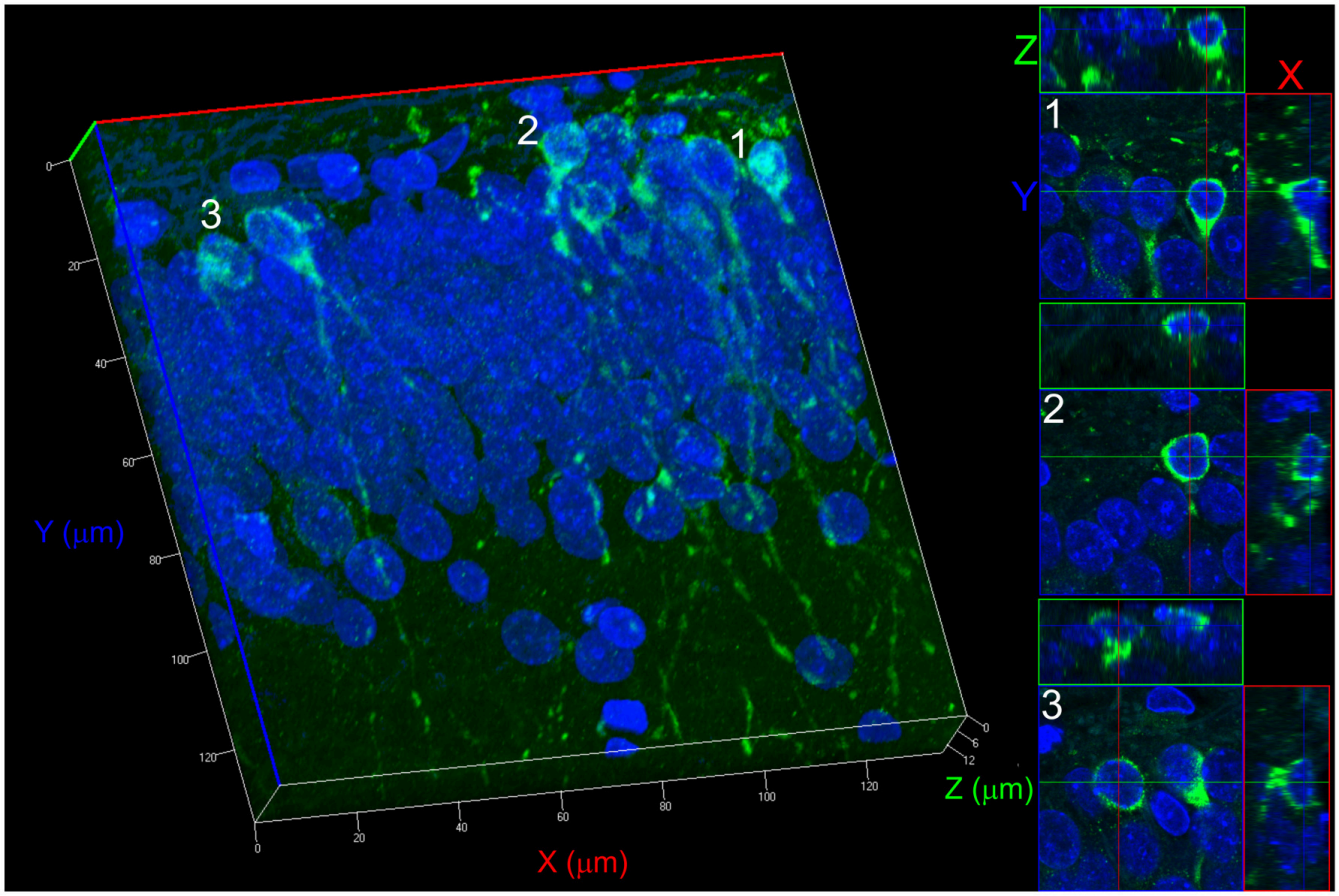
**3D reconstruction process for counting newborn neurons**. The example shown refers to a Control group. The reconstruction was performed with 15 confocal z-stacks, 0.8 μm increments, pinhole adjusted to 1 Airy unit, with a 63× oil-immersion objective. The projections on the X, Y, and Z planes allow determining the non-overlapping positioning of two cells from two different confocal optical sections, avoiding a misidentification of morphology type. Three different cell projections (1, 2, and 3) are shown on the right side demonstrating how cells can be seen for identification and counting.

**Figure 2:**
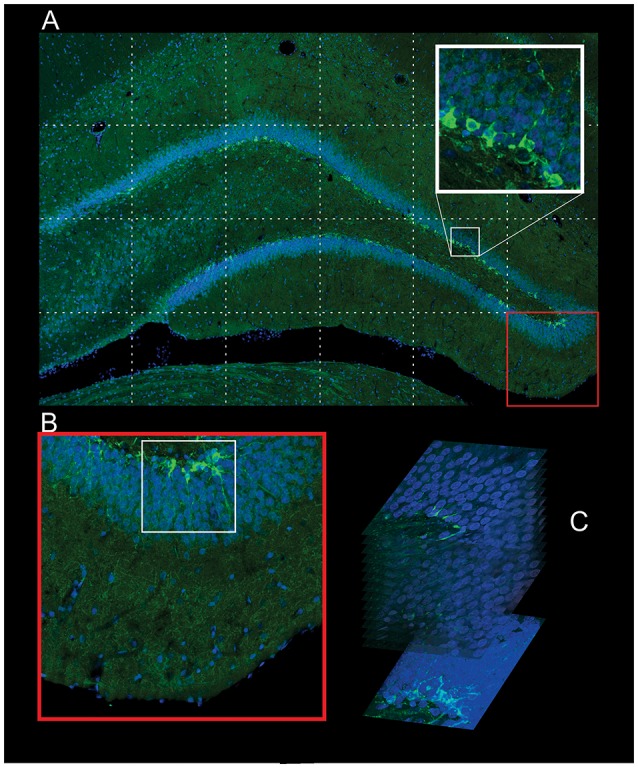
**Procedure used in the identification of the newborn neurons. (A)** Confocal Optical Section (COS) image of the whole DG in high resolution (24 megapixels) composed with several pictures captured using the 20× objective. The high-resolution of the COS is enough to identify the morphology of the DCX+ neurons and also to count (red square). **(B)** The COS image is used to monitor the regions (white square, bottom left) that will be captured with 63× objective in different z-stacks, 0.8 increments. **(C)** The summation of these stacks gives the image used to identify the morphology type of the newborn neuron (bottom right).

To assess the number, morphology and location of newborn granule cells, confocal optical sections (COS) of the entire DCX-labeled dentate gyrus were used to count the DCX+ neurons. Imaging was performed using a Zeiss LSM710 confocal system set up on an Observer-Z1 inverted microscope with a 20× objective (numerical aperture 0.50). All images were captured with identical confocal settings for each animal (excitation wavelength, 488 nm; the same power settings; emission range collected, 493–586 nm). From the captured images (frames) a reconstruction of the entire area of the dentate gyrus was made, creating the image of 6,000 × 4,000 pixels (24 megapixels). This image is a COS. Three COS per histological section and 6 histological section per rat were used in the quantification, resulting a total of 18 COS per rat. From each hippocampus 3 sections were taken, one 600 μm apart from each other. Three COS images were captured per histological section. The first COS was captured centered at 7 μm, the next at 19 μm and the third at 31 μm. According to Wojtowicz and Kee (2006), when counting of DCX+ cells, due to the small number of cells, usually 80–100 cells per section, a fewer sections per hippocampus are sufficient. The histological sections were obtained in the coronal plane, from rostral to caudal, from the middle portion of the hippocampus, from AP: −2.6 to −4.6 mm, having the bregma suture as reference, using the Paxinos and Watson (2007) stereotaxic Rat Brain Atlas. Once the middle portion of the hippocampus was determined, the intermediate slice was chosen, identifying the one whose anatomical structures were the closest to those presented in the Atlas at AP: −3.6 mm. The other two were chosen by taking the nearest slice of AP = −3.0 mm and the nearest slice of AP = −4.2 mm. The DCX+ neurons of the COS were manually identified, classified and counted using a program developed in Matlab platform. When necessary, using the ZEN 2010b software (Zeiss, Jena, Thuringia, Germany), the morphology and location of the stained neurons were determined using a three-dimensional reconstruction using 15 confocal z-stacks, 0.8 μm increments, pinhole adjusted to 1 Airy unit, with a 63× oil-immersion objective (Figures 1, 2). All cells DCX+ are marked with the “position” function of the ZEN 2010b (Zeiss, Jena, Thuringia, Germany). This function saves the (x,y,z) coordinates. When it is not clear the pertinence of the cell to the optical section, like in the situation of lost caps, or when it is not clear the morphology of the cell, with these coordinates the Software is able to reconstruct the 3D image (63x objective) centered in the corresponding coordinates.

### Statistical analysis

We compared control rats with epileptic rats and with epileptic rats subjected to physical exercise. The average number of the different types of DCX+ neurons investigated per COS (n/cos) was the variables analyzed. We used the Shapiro-Wilk normality test to verify the normality of the data. Quantitative variables are reported as mean ± standard deviation (SD) and were compared using One-way analysis of variance (ANOVA) tests and Tukey's multiple comparison tests when the variable analyzed was compared between more than two groups. When the comparison involved only two groups, it was used Student's *t*-test. A *P* < 0.05 was considered as significant.

## Results

The number, location, and morphology of the newborn neurons in the dentate gyrus were assessed in each animal with DCX immunohistochemistry (Brown et al., 2003; Couillard-Despres et al., 2005; Wojtowicz and Kee, 2006). Samples were first analyzed for the presence of cells that showed fluorescent labeling for DCX and these were named DCX+ neurons. As shown in **Figure 4A**, the SE group exhibited fewer DCX+ neurons than the rats in the C and SE/EX groups. The average number of DCX+ neurons per COS in the chronic pilocarpine-treated epileptic rats subjected to physical exercise was not significantly different from the number observed in the C group (DCX+ C: 65.51 ± 3.94 n/cos, SE: 43.94 ± 5.21 n/cos, SE/EX: 61.85 ± 8.40 n/cos).

**Figure 3:**
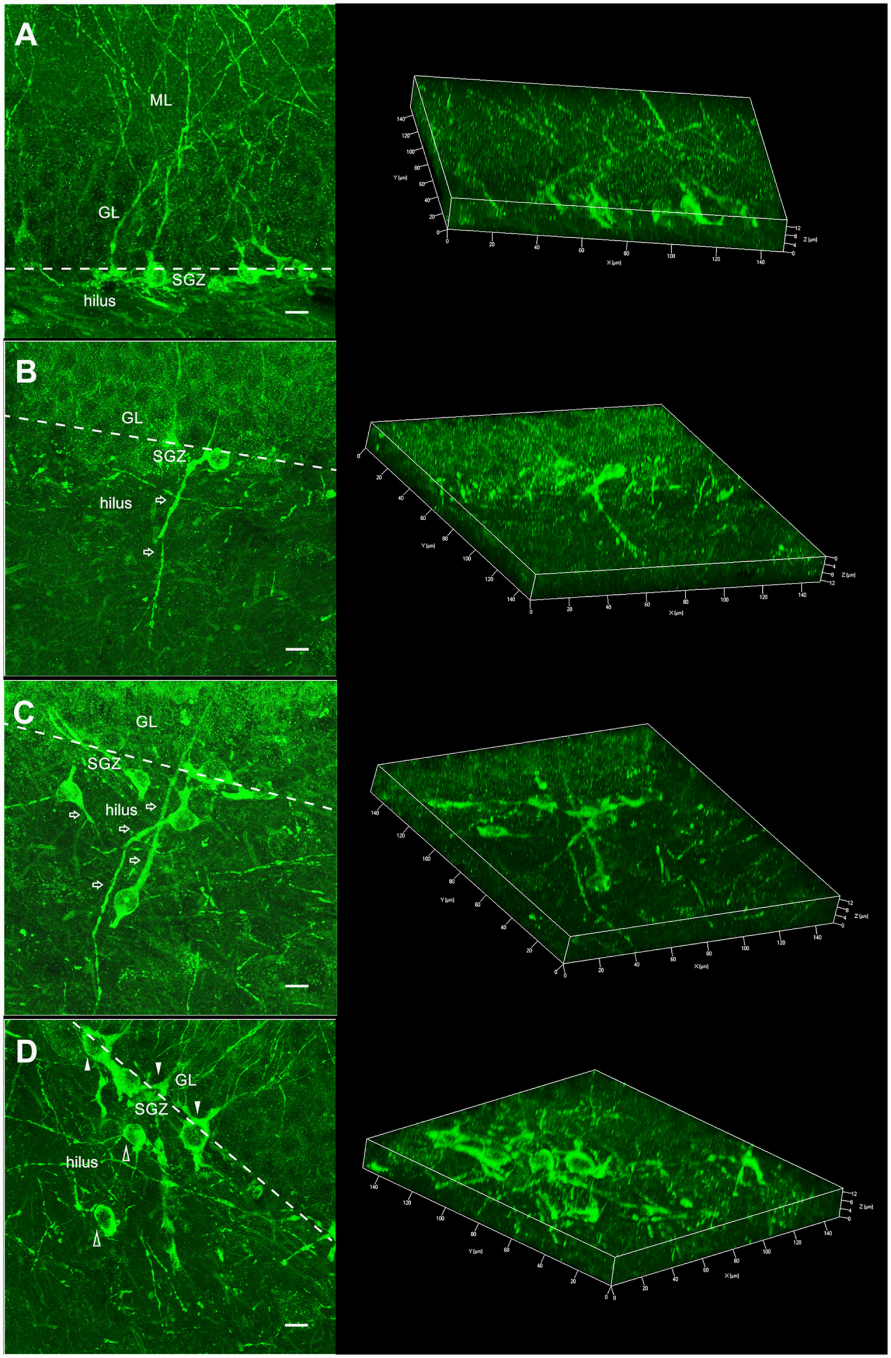
**Morphology of the DCX+ neurons showed in two different views: summation of several confocal optical sections allowing to view the complete morphology of the newborn neuronal (left) and 3D reconstruction where the confocal optical sections were rendered and the spatial morphology of the newborn neuronal can be inspected from different visions (right). (A)** DCX+ neuron with regular localization and morphology: cell body in the dentate gyrus granule/subgranule layer and apical dendrite toward the ML/perforant path, typical of the C group (nrDCX+). **(B)** DCX+ normotopic neuron with basal dendrites reaching deep into the hilus (nbdDCX+). This morphology was observed in the SE and SE/EX groups. **(C)** DCX+ ectopic neurons (ehDCX+) localized in the hilus and with aberrant dendrites (open arrows). This characteristic was observed in the SE and SE/EX groups. **(D)** nrDCX+ neurons (filled arrowheads) and ehDCX+ neurons (open arrowheads) in the same image, a common characteristic observed in the SE/EX group (ML, molecular layer; GL, granule layer; SGZ, subgranule zone; bar, 10 μm).

**Figure 4:**
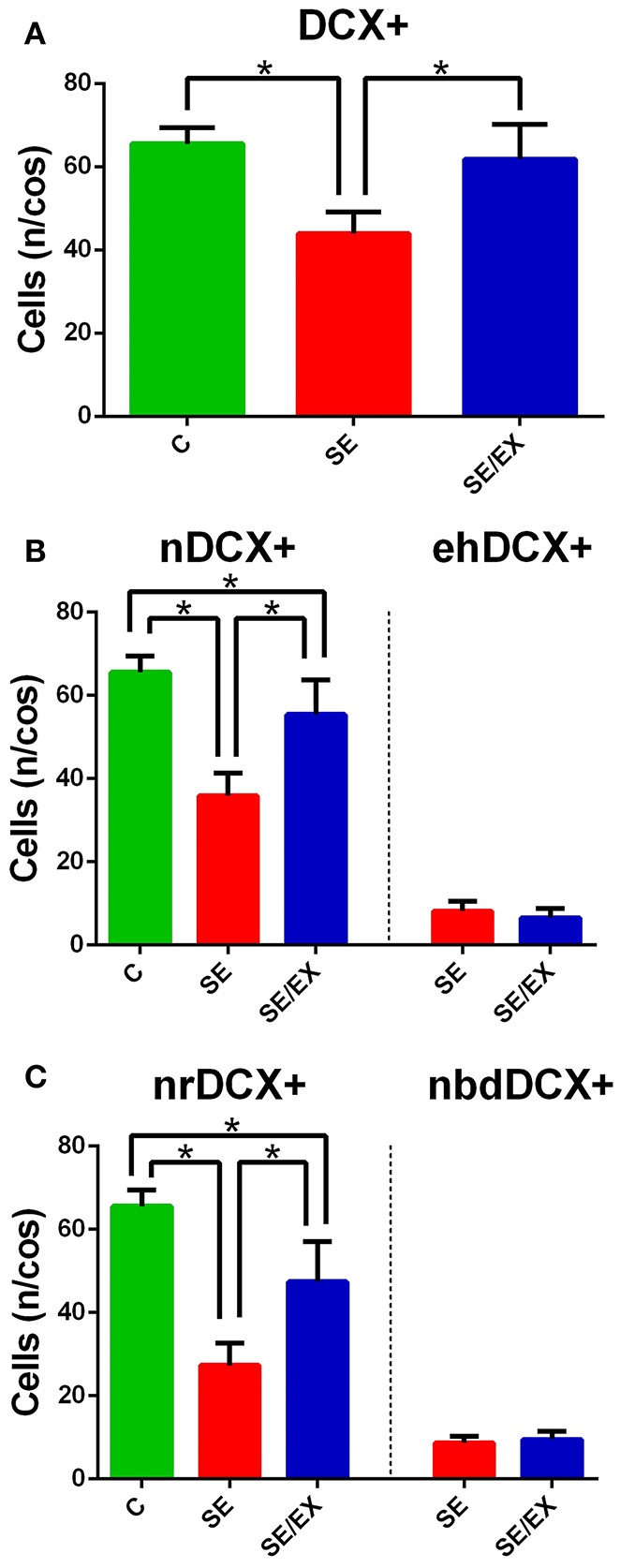
**Statistical comparison of the DCX+ stained neurons in the DG for the groups C, SE, and SE/EX groups. (A)** The average number of DCX+ stained neurons per confocal optical section is lower in the SE group. **(B)** The average number of normotopic DCX+ stained neurons is lower in the SE group. There was no statistically significant difference of the average number of ectopic hilar DCX+ stained neurons per confocal optical section between the groups SE and SE/EX. **(C)** The average number of normotopic regular DCX+ stained neurons per confocal optical section is lower in the group SE. The average number of normotopic neuron with basal dendrites reaching deep into the hilus DCX+ stained neurons was not different comparing SE and SE/EX groups. ^*^p < 0.05 a One-way ANOVA and Tukey's *Post-hoc* for DCX+, nDCX+, nrDCX+. ^*^p < 0.05 a Student's *t*-test for ehDCX+, nbdDCX+. Data are expressed in mean ± SD.

Next, DCX+ neurons were investigated for targeting and classified into two groups. DCX+ neurons with the cell body regularly located in the granule layer were classified as normotopic newborn neurons (nDCX+). DCX+ neurons ectopically located in the hilus were classified as abnormal newborn neurons ectopically located in the hilus (ehDCX+). In Figure 3, the migration targeting of the newborn neurons on the C, SE and SE/EX groups are shown. Comparing the groups for the location of the newborn neurons, the average number of nDCX+ (n/cos) was different in the three groups (higher in the group C, followed by SE/EX and lower in the group SE) (nDCX+ C: 65.51 ± 3.94 n/cos, SE: 35.85 ± 5.429 n/cos, SE/EX: 55.35 ± 8.35 n/cos; Figure 4B). There was no statistically significant difference between the groups SE e SE/EX for the variable ehDCX+ (ehDCX+ SE: 8.10 ± 2.35 n/cos, SE/EX: 6.50 ± 2.28 n/cos; Figure 4B).

In the next step, the nDCX+ neurons were analyzed for the occurrence of deep basal dendrite in the hilus. nDCX+ neurons with no basal dendritic projecting into the dentate hilus were classified as normotopic regular newborn neurons (nrDCX+). nDCX+ neurons located in the granule layer, but with basal dendrites projecting to the hilus were classified as normotopically located newborn neurons with basal dendrites projecting into the dentate hilus (nbdDCX+). Figure 3B shows a neuron with a basal dendrite. The three-dimensional reconstruction images in Figure 3 were formed from 15 confocal image stacks through the z-depth, allowing identification of the basal dendrites in newborn neurons stained with DCX. There was no statistically significant difference between the SE and SE / EX groups in the variable nbdDCX+ (nbdDCX+ SE: 8.61 ± 1.64 n/cos, SE/EX: 9.42 ± 1.93 n/cos). The average number of nrDCX+ neurons per COS in the SE/EX rats was significantly lower than the number in rats from the C group and greater than the number in the SE rats (Figure 4C; nrDCX+ C: 65.51± 3.94 n/cos, SE: 27.24 ± 5.32 n/cos, SE/EX: 47.38 ± 9.62 n/cos).

In order to compare the data reported in the literature on abnormal neurons in animal models of chronic epilepsy (Jessberger et al., 2007b; Walter et al., 2007), the ehDCX+ neurons and the nbdDCX+ neurons were grouped into a single group termed as abnormal DCX+ neurons. Almost half of the DCX+ neurons in the SE rats presented abnormalities in localization and/or presence of basal dendrites, 16.70 ± 3.25 abnormal n/cos out of 43.94 ± 5.21 total DCX+ n/cos. In the SE/EX group, 15.92 ± 3.54 abnormal n/cos out 61.85 ± 8.40 of total n/cos of the DCX+ neurons exhibited abnormalities. However, the number of abnormal DCX+ neurons per COS was not significantly different between the SE and SE/EX groups.

## Discussion

Recent findings have converged on the hypothesis that the abnormal integration of adult-generated newborn dentate granule cells has effect on the development of TLE (Parent and Lowenstein, 2002; Hester and Danzer, 2013; Althaus et al., 2015). It has also been demonstrated that physical exercise promotes adult hippocampal neurogenesis (Cotman and Berchtold, 2002; Fabel et al., 2003; Lee et al., 2013; Nokia et al., 2016). Although decreased neurogenesis is not causally associated with epilepsy and is, in fact, an effect of the disease, it is reasonable to suspect that the beneficial effects of physical exercise on the comorbidities associated with epilepsy (Roth et al., 1994; Gobbo and O'mara, 2005; Arida et al., 2012; Gomes et al., 2014) might be due to changes in the neurogenesis processes (Hattiangady and Shetty, 2008). To test this prediction and evaluate the neuronal changes, we determined the number, location and morphology of the newborn neurons in the dentate gyrus by comparing anti-DCX staining in pilocarpine-treated epileptic rats subjected and not subjected to physical exercise and also control rats.

The present study revealed a significant reduction in the generation of newborn granular cells in epileptic rats compared to control rats. Despite the nonconventional counting protocol used, where the density of DCX+ neurons per section were evaluated, and not an estimate of the total number of DCX+ neurons in the hippocampus as usually is performed using stereological sampling, the measure was able to quantify significant changes that were consistently shown in the slices of all animals investigated. The results are in agreement with previous demonstrations of reduced hippocampal neurogenesis in the chronic phase of the disease (Hattiangady et al., 2004; Walter et al., 2007). The disease-induced changes in micro-environment, such as reduced levels of neurotrophic factors (FGF-2, IGF-1, and BDNF), observed in chronic epileptic hippocampi, and the non-neuronal fate-choice decision of newly born cells, are being implicated as the main cause of the reduced neurogenesis (Shetty et al., 2003; Hattiangady et al., 2004; Hattiangady and Shetty, 2008, 2010). The reduction in the generation of newborn granule cells observed in the epileptic group was not observed in the epileptic group subjected to physical exercise. Therefore, besides the cellular proliferation as described in Gomes et al. (2014), the present findings suggest that physical exercise could be able to affect the neuronal differentiation of newly born cells in the chronically epileptic hippocampus. The main effect may be a positive contribution to the neurogenesis, counteracting the neurodegeneration associated with epilepsy.

Despite the absence of changes in the number of abnormal newborn neurons observed, the present data show that physical exercise is able to promote an increase in the number of regular newborn neurons. Many studies had reported the beneficial effects of physical exercise in decreasing the frequency and severity of seizures (Denio et al., 1989; Eriksen et al., 1994; Arida et al., 1998, 1999, 2003, 2007; McAuley et al., 2001; Gomes et al., 2014). According to Walter et al. (2007), newborn granule neurons may be more vulnerable to environmental disturbances than mature neighboring neurons. Therefore, the initial SE insult as well as the occurrence of spontaneous seizures may affect the migration and maturation processes, leading to both the occurrence of hilar ectopic neurons as well as hilar basal dendrites on the normotopic neurons (Jessberger et al., 2007a,b; Walter et al., 2007). Indeed, taking into account the expected recursive interplay of the effects, the occurrence of fewer seizures may result in fewer abnormalities in the newborn neurons induced by physical exercise.

The positive effects of physical exercise on mood disorders in subjects with epilepsy may be reinterpreted with the present findings. Disruption of the neurogenesis induced by antidepressant drugs blocks the behavioral improvements due to these drugs (Malberg et al., 2000; Manji et al., 2001; Nestler et al., 2002; Santarelli et al., 2003; Drew and Hen, 2007; Sahay and Hen, 2007; David et al., 2009). Once antidepressant drugs induce normal neurogenesis, the beneficial effects of physical exercises in epilepsy, particularly in reducing depression (Roth et al., 1994; Arida et al., 2012; de Lima et al., 2013), may be reinterpreted taking into account the increase in the number of newborn neurons revealed in our data.

Another comorbidity of epilepsy that is positively affected by physical exercise is cognitive impairment (Gobbo and O'mara, 2005; Gomes et al., 2014). Our data may also contribute to the understanding of this effect. According to Cho et al. (2015), the cognitive dysfunction resulting from pilocarpine induced-SE is associated with the neurogenesis of ectopic neurons. This association was also observed in other models of epilepsy (Jessberger et al., 2007a; Scharfman and Pierce, 2012) and the interpretation is that the ectopic neurons may contribute to sustaining epileptiform activity (Parent, 2007), thus they may be more crucial to functional alteration following SE than neurogenesis in a brain not injured. Evidences have been collected sustaining the role of the newborn neurons in the DG of the hippocampus improving or maintaining learning and memory functions (van Praag et al., 1999; Shors et al., 2002; van Praag et al., 2002; Drapeau et al., 2003; Cao et al., 2006; Uda et al., 2006). Therefore, although our findings show that physical exercise does not reduce the number of ectopic newborn neurons, the increase in the number of normotopic regular newborn neurons could counteract the effect of the ectopic neurons, which may contribute for reducing cognitive impairment. Further investigations must be conducted to support this hypothesis. Our findings motivate the design of new investigations focused on the survival and functionality of newborn neurons, paving the way to unravel the mechanisms responsible for the beneficial effects of physical exercise on epilepsy comorbidities.

## Author contributions

FM: carried out the experiments, interpreted the data and collaborated in the manuscript writing. LS: performed the confocal microscopy images. AR: analyzed the data and discussed the manuscript writing. SG, RA, GD, and FS: discussed the results and the manuscript writing. A-CA: designed experiments interpreted the data and wrote the manuscript.

### Conflict of interest statement

The authors declare that the research was conducted in the absence of any commercial or financial relationships that could be construed as a potential conflict of interest.
